# Cost patterns of long-term prescribed medications in patients with multiple sclerosis in Germany

**DOI:** 10.1038/s41598-025-25318-4

**Published:** 2025-10-27

**Authors:** Michael Hecker, Birgit Berger, Niklas Frahm, Felicita Heidler, Bassel Barhoum, Avinash Mohnish Suntah, Finn Brüggemann, Matthias Grothe, Uwe Klaus Zettl

**Affiliations:** 1 Division of Neuroimmunology, Department of Neurology, Rostock University Medical Center, Rostock, Germany; 2 Ecumenic Hainich Hospital gGmbH, Mühlhausen, Germany; 3 Department of Neurology, University Medicine Greifswald, Greifswald, Germany

**Keywords:** Multiple sclerosis, Drug prescriptions, Medication costs, Germany, Multiple sclerosis, Drug therapy

## Abstract

Multiple sclerosis (MS) is a chronic neurological disease that affects people in their most productive years of life. A multimodal treatment approach is typically employed to slow the progression of disability and alleviate MS-related symptoms. This implies a high financial burden of MS on patients, healthcare systems and society. Our study focused on the costs that are attributable to long-term drug prescriptions in patients with MS. For this purpose, the medication plans of 728 MS patients from 3 medical centers in Germany were analyzed. Pharmaceutical pricing information was obtained from the LAUER-TAXE database in May 2024. The costs for the therapy with disease-modifying drugs (DMDs), the treatment of symptoms and the drug management of comorbidities were calculated separately. We then explored how the annual medication costs are related to clinical and demographic characteristics of the patients. Apart from the use of DMDs (*n* = 584 patients, 80.2%), an average of 2.9 other prescribed medications were taken by the patients on a long-term basis. The annual medication costs averaged €11,788. DMDs contributed to 92.1% of the cumulative costs and explained 25.36% of the variance in total costs alone. The total costs were higher in younger patients with relapsing MS and mild to moderate disability due to their more frequent use of expensive DMDs. The costs for symptomatic medications and comorbidity medications increased with age, degree of disability and number of comorbidities. However, the large variability in the costs for individual patients could only be partly explained by regression models. Our study provides current data on the costs of prescribed medications, which are a major direct cost element in the care of MS patients. The clinical heterogeneity of the patients is reflected in a great variety in drug consumption and in a broad distribution of medication costs. The highest medication costs are incurred before the age of 50, as an early effective treatment can minimize later indirect costs of MS.

## Introduction

Multiple sclerosis (MS) is a chronic immune-mediated disease of the central nervous system^1–3^. The disease is characterized by demyelination and neuro-axonal damage in the brain and spinal cord, which leads to a wide range of symptoms, such as fatigue, motor dysfunction, sensory issues, visual problems and cognitive impairment^4–6^. MS is usually diagnosed between the ages of 20 and 40, affecting approximately 2.9 million people worldwide and around 280,000 individuals in Germany alone^7^. MS is thus the most common neurological condition among people of working age, and it requires lifelong treatment to mitigate symptoms and prevent the accumulation of permanent neurologic disability. Disease-modifying drugs (DMDs) form a central component of long-term MS therapy, and they differ in their mechanisms of action as well as routes of administration (oral, injection or infusion)^8–12^. However, the treatment of MS consists of a multidisciplinary approach that, in addition to DMDs, also includes acute relapse treatment^13^, symptom control^4^, comorbidity management, psychological support, rehabilitation interventions and lifestyle modifications^8^. The socioeconomic burden of MS is thus substantial.

Disease-related costs can be divided into direct costs and indirect costs. Direct costs comprise expenses directly related to medical care (i.e., direct medical costs), e.g., for medications, hospital stays, diagnostic tests and rehabilitation services, as well as expenses related to the disease (i.e., direct non-medical costs), e.g., for transportation, walking aids, home modifications and support services. Indirect costs primarily refer to productivity losses due to absence from work and early retirement. MS imposes particularly high direct and indirect costs on patients, caregivers and society due to the chronic nature of the disease and the ongoing need for medical care^14–17^.

High and rising prices of DMDs, independent of their route of administration, are a major component of healthcare expenditures in the treatment of MS^18^. In Germany, the pricing and reimbursement of drugs is regulated by a combination of government policies, health insurance funds and independent institutions. This process involves benefit assessments and price negotiations with pharmaceutical manufacturers to ensure that patients have access to necessary medicines while keeping healthcare costs under control^19^. The outcome is the statutory retail price of drugs, which aims to balance the needs of pharmaceutical companies, insurance providers, pharmacies and patients. A co-payment (usually a maximum of €10) is charged to patients in Germany for each prescription (Rx)^19^. In addition, many patients bear out-of-pocket expenses for non-reimbursed and lifestyle medications^20,21^. For the inpatient setting, a separate reimbursement mechanism has been in place in Germany, the so-called Diagnosis-Related Groups system, according to which a fixed amount was paid to hospitals to cover all services and treatments^19,22^, but major reforms were initiated in 2025^23^.

Previous studies estimated the total costs of MS in Germany at ~€20,000 to ~€60,000 per year, with disability being the key cost driver^24–26^. In patients with a high degree of disability, the direct non-medical costs and indirect costs are much higher than the direct medical costs, as reviewed elsewhere^14^. The cost for the therapy with DMDs alone represent around 55–70% of the total costs for patients with mild disability and around 5–20% of the total costs for patients with severe disability^24–26^. Differences in the distribution of costs were also shown for the different courses of MS^27^: In comparison to patients with relapsing-remitting MS (RRMS), drug costs are lower for patients with primary progressive MS (PPMS) or secondary progressive MS (SPMS)^26,28,29^, reflecting that the treatment options for progressive MS are still limited^30^. In fact, ocrelizumab is the only approved DMD for PPMS, while its use in Europe is restricted to patients with evidence of inflammatory disease activity^30,31^. Over the last years, the rates of symptomatic treatment have increased for the most common MS-related symptoms^4,32^, contributing to increased resource utilization and healthcare costs. Another cost component is the need for medication to treat comorbidities, which plays an increasing role as the average age of MS patients is rising^33^.

Studies on medication costs in patients with MS vary in design, methodology and data sources, making it difficult to compare findings across studies. They are often based on patient self-reporting and usually do not consider the costs for comorbidity drugs. The rapidly changing treatment landscape requires up-to-date analyses, but only a few studies on the costs of MS in Germany have been published since 2019: Ness et al. have studied specifically the costs of relapse^34^ and the costs of DMDs but not other prescribed medications^35^ for patients who were treated with selected DMDs. The only other recent study is by Dillon et al. who determined the costs for DMDs and a range of other medications using data from outpatient neurology clinics^24^.

The aim of this study was to analyze the costs of all long-term prescribed medications, including medications for the treatment of symptoms and comorbidities, in a large group of patients with MS. We sought to assess annual per-patient medication costs based on drug prices in Germany in 2024. On this basis, we aimed to gain closer insights into the distribution of these costs and their association with the patients’ degree of disability and other patient-specific factors.

## Methods

### Study population

**Fig. 1:**
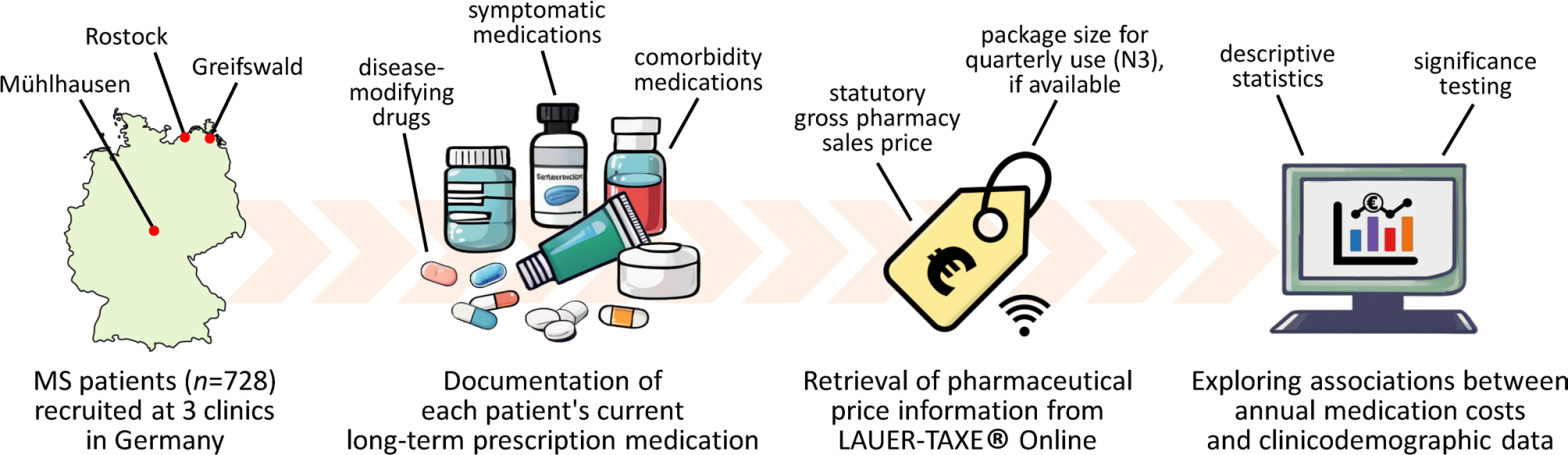
Workflow of the study. The medication plans of a total of 728 patients with multiple sclerosis (MS) were collected between March 2017 and June 2022. More specifically, we recorded the brand name, route of administration, dosage and application frequency of each long-term prescription medication being used. Drug prices were gathered in May 2024 from LAUER-TAXE Online^49^, a database for healthcare professionals that provides pricing information and reimbursement rates for pharmaceutical products available in Germany. We then determined the patients’ individual annual medication costs in € and analyzed the contributing factors.

This study followed a cross-sectional design and involved 728 patients. The patients were recruited at the Departments of Neurology of 3 medical centers in Germany: University Medical Center Rostock, Ecumenic Hainich Hospital Mühlhausen and University Medicine Greifswald (Fig. 1). The patients were asked to participate in the study either after outpatient appointments or during inpatient hospital stays. Inclusion criteria were an age of ≥ 18 years and a clinically isolated syndrome (CIS) suggestive of MS or a confirmed diagnosis of MS according to the 2017 revisions of the McDonald criteria^36^. Routine medical care was provided to all patients. Accordingly, the patients were treated and monitored following the guidelines and recommendations of the German Society of Neurology^31^. These guidelines are based on clinical evidence regarding efficacy and safety, while drug costs and cost-effectiveness are generally not considered.

The study was approved by the ethics committees of the University of Rostock and of the State Medical Association of Thuringia (permit numbers A 2014-0089 and A 2019-0048). Our study was conducted according to the Declaration of Helsinki, the Good Clinical Practice Guidelines and the European General Data Protection Regulation. All patients participated voluntarily and provided written informed consent in advance.

### Data collection

Sociodemographic, clinical and medication data of the 728 patients were collected between March 2017 and June 2022. The data were gathered for each patient through a review of medical records, a clinical examination and a structured interview. The sociodemographic data included age, sex, place of residence (< 5,000 residents: rural community, 5,000–19,999: provincial town, 20,000–99,999: medium-sized town, ≥ 100,000: city), years of schooling, educational level, employment status and partnership status. The clinical data comprised the type of patient care received as well as the patient’s disease course (CIS/RRMS, SPMS or PPMS)^27^, clinical disability, disease duration and comorbidities. The degree of disability was assessed using the Expanded Disability Status Scale (EDSS), which ranges from 0 (normal neurological status) to 10 (death due to MS)^37^. Comorbidity was defined as any coexisting disorder that developed before or during the course of MS^38^. The medication data covered all prescribed medications for long-term use. For this purpose, the medication schedule of each patient was examined to retrieve brand name, active ingredient, dosage, drug formulation, route of administration, application frequency and indication for every drug received.

### Drug classification

The medications were categorized by the treatment goal into DMDs, drugs to relieve MS-related symptoms and drugs to treat comorbidities or other conditions (Fig. 1), as in our previous studies^39,40^. The DMDs for MS are primarily those that are approved for MS according to the European Medicines Agency labels^1,10,31^. Moreover, the DMD category also included drugs that were sometimes used off-label for the treatment of MS, namely rituximab, an anti-CD20 monoclonal antibody^41^, intravenous immunoglobulins (IVIg), which were at times administered in young women who were pregnant or planning to become pregnant^42–44^, and glucocorticosteroids (GCs), when used as repeated pulse therapy (usually every 3 months) for progressive courses of MS^45,46^. Symptomatic medications included, for instance, fampridine to improve walking ability, nabiximols to alleviate spasticity and oxybutynin to treat overactive bladder. All the other medications that were prescribed for secondary conditions, e.g., osteoporosis, hypertension or gastrointestinal problems, were subsumed as comorbidity medications. Rx polypharmacy was defined as being on five or more Rx drugs on a long-term basis^47^.

For the present analysis, we only considered Rx medications that were permanently taken in regular intervals, independently of whether the patients were outpatients or inpatients at the time of data collection. Therefore, we excluded on-demand medications that were taken irregularly as needed or in acute circumstances, such as GCs for the treatment of an MS relapse. We also excluded over-the-counter (OTC) medications, which are purchased directly by the patients. However, some medications that do not require a prescription can be prescribed in Germany at the expense of the statutory health insurance according to the OTC exception list and were therefore included (e.g., vitamin D preparations)^48^.

### Medication cost calculation

The annual costs were calculated in euros (€) for every drug that had been prescribed to a patient on a long-term basis. We used LAUER-TAXE Online 4.0, which is a central database for the German healthcare system that contains prices for medicines and other pharmaceutical products^49^. The statutory retail prices as of May 2024 were used. These specify the price of a medicine including statutory surcharges and value-added tax, i.e., the retail price in pharmacies (referred to as GES. VK or TAXE-VK in the software). The actual costs for the health insurance are lower than this, because rebates (e.g., due to confidential agreements with the pharmaceutical companies) and statutory co-payments by the patients are not yet subtracted from this price. LAUER-TAXE shows multiple prices per medication if there are different distributors or if the medication is sold in different formulations, dosages and pack sizes. In Germany, a distinction is made between N1, N2 and N3 as standardized size classes for pharmaceutical packages^50^. N1 stands for a small pack for acute treatments or for testing a drug. N2 is a medium-sized pack and lasts for ~ 30 days (monthly demand). N3 is intended for long-term therapy and lasts for ~ 100 days (quarterly demand). It should be noted that historical price data was inaccessible, thus price fluctuations over time since the medication data was collected could not be accounted for.

For the cost calculation, we have used the lowest price if there are different distributors. If the medication was taken in the standard dose, we usually multiplied the lowest price for N3 packs by 4 to estimate annual costs, assuming quarterly dispensing. If a patient took the medication more or less often (e.g., every other day instead of daily), the price for the bulk pack was multiplied by a correspondingly larger or smaller factor. In the case of infrequent drug use, only the portion of an N3 pack that was actually consumed per year was multiplied by the retail price. It was also taken into account that the administered dose may deviate from the standard dose in individual cases. For example, a patient may only take half a tablet instead of a whole tablet per day, which halves the number of packs required per year. If no N3 pack is sold for a medication, the annual costs were calculated on the basis of a smaller pack that would allow the most cost-effective therapy at the corresponding dosage and application frequency. If it was not possible to find out the dose of a medication used by a patient, the most favorable annual price was determined (e.g., 4 times the N3 pack with the lowest dose). This was especially the case for medicines that are administered in the form of liquids, sprays or creams. If there was no price information for a medication in LAUER-TAXE (e.g., for articles that are no longer distributed), the price was set to €0. Finally, the annual prices were summed up over all medications and per medication category for each patient. The final costs have been rounded to whole €.

### Statistical analysis

The patient data, medication schedules and price tables were compiled in IBM SPSS Statistics 27.0 and Microsoft Excel 2010. The calculation of medication costs and the statistical analysis of the data were carried out in R version 4.1.2.

Descriptive statistics of sociodemographic, clinical and medication data as well as medication costs were determined as means, standard deviations (SD), standard errors, medians, ranges, frequencies and percentages. A histogram and bar charts were used to visualize the distribution of drug prescriptions and costs. The average annual medication costs were calculated for the entire patient population as well as for subgroups. The patient subgroups were defined on the basis of categorical characteristics and compared using Mann-Whitney *U* tests or Kruskal-Wallis tests. The significance level was set at *α* = 0.05. Results were considered highly significant if *p* < 0.001.

The association between patient data and medication costs was explored using correlation analyses and regression models. Kendall’s *τ* was used to assess the correlation between variables. The sociodemographic, clinical and medication data were considered separately in different linear models. The relative importance of individual regressors was then assessed using the R package relaimpo^51^. This was done using the lmg metric, which provides a decomposition of the variance explained by the model into non-negative contributions^52^. Generalized additive models were used to fit smooth curves with 95% confidence intervals to visualize the relationship between age and annual medication costs. The deviance explained (DE) by the models was used as a measure to evaluate how well age and EDSS score account for the variability in costs.

## Results

### Patient characteristics

A total of 728 patients with MS were included in this study (Table 1). The patients were on average 48.6 ± 13.2 (mean ± SD) years old. The female-to-male ratio was 2.5:1. Half of the patients (*n* = 364) were already retired due to age or disability, and the majority of patients were living in a partnership (*n* = 528, 72.5%). The patient cohort was composed of cases with CIS/RRMS (*n* = 483, 66.3%), SPMS (*n* = 176, 24.2%) and PPMS (*n* = 69, 9.5%). Most patients were treated as outpatients when they were included in the study (*n* = 493, 67.7%). The average EDSS score of the patients was 3.6 ± 2.1 (range: 0–9) at a median disease duration of 10 years (range: 0–52). In addition to MS, the patients typically had one (*n* = 173, 23.8%) or more (*n* = 350, 48.1%) comorbidities. Older age was significantly associated with a higher degree of disability (*τ* = 0.341) and a higher number of comorbidities (*τ* = 0.266) (*p* < 0.001). The patients with CIS/RRMS had a lower mean EDSS score (2.6 vs. 5.7) and were more often employed (54.0% vs. 15.5%) than patients with progressive MS. Most of them were also treated as outpatients (84.5%), while the percentage of outpatients was much lower for SPMS (34.7%) and PPMS (34.8%).

**Table 1:** Overview of the MS patient cohort (*n* = 728).

Characteristic	Statistics	Characteristic	Statistics
**Sociodemographic data**		**Clinical data**	
Age (years), mean ± SD	48.6 ± 13.2	Medical center, *n* (%)	
Sex, *n* (%)		Rostock	421 (57.8)
Women	519 (71.3)	Mühlhausen	206 (28.3)
Men	209 (28.7)	Greifswald	101 (13.9)
Place of residence, *n* (%)		Patient care, *n* (%)	
Rural area	274 (37.6)	Outpatient	493 (67.7)
Provincial town	125 (17.2)	Inpatient	235 (32.3)
Medium-sized town	146 (20.1)	Disease course, *n* (%)	
City	183 (25.1)	CIS/RRMS	483 (66.3)
School years, median (range)	10 (6–18)	SPMS	176 (24.2)
Educational level, *n* (%)		PPMS	69 (9.5)
No training	27 (3.7)	EDSS score, mean ± SD	3.6 ± 2.1
Skilled worker	459 (63.0)	Disease duration (years), median (range)	10 (0–52)
Technical college	105 (14.4)	Number of comorbidities, median (range)	1 (0–9)
University	137 (18.8)		
Employment status, *n* (%)			
Employed	299 (41.1)	**Medication data**	
Unemployed	31 (4.3)	Long-term Rx medications, mean ± SD	3.7 ± 2.7
Retired	364 (50.0)	DMD use, *n* (%)	
Other	34 (4.7)	Yes	584 (80.2)
Living in a partnership, *n* (%)		No	144 (19.8)
Yes	528 (72.5)	Symptomatic Rx medications, mean ± SD	1.2 ± 1.5
No	200 (27.5)	Comorbidity Rx medications, mean ± SD	1.7 ± 2.0

CIS = clinically isolated syndrome, DMD = disease-modifying drug, EDSS = Expanded Disability Status Scale, MS = multiple sclerosis, PPMS = primary progressive multiple sclerosis, RRMS = relapsing-remitting multiple sclerosis, Rx = prescription, SD = standard deviation, SPMS = secondary progressive multiple sclerosis.

### Drug prescriptions and annual medication costs

**Fig. 2:**
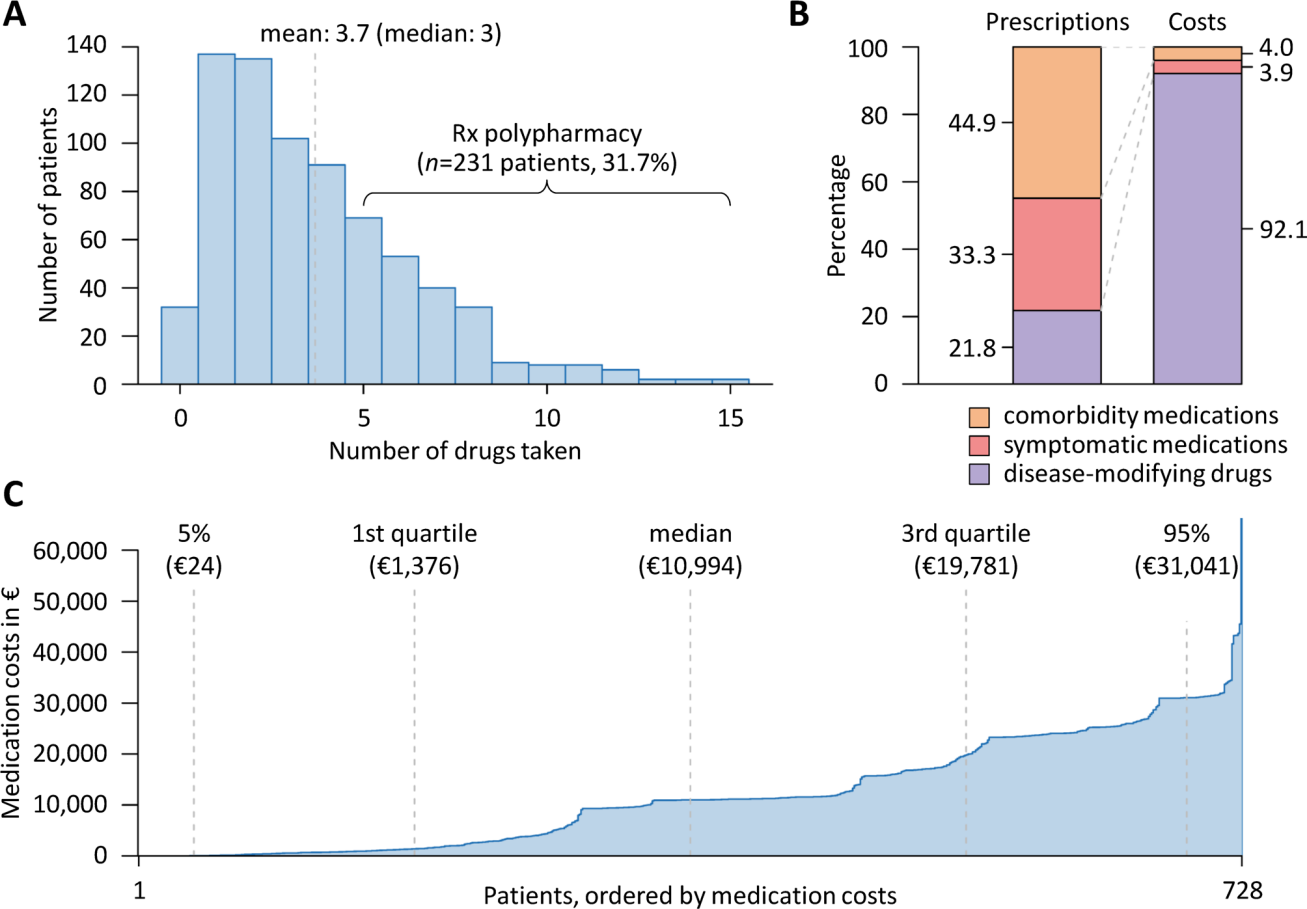
Distribution of drug prescriptions and annual medication costs. (**A**) For the 728 patients with multiple sclerosis (MS), a total of 2,681 long-term drug prescriptions (Rx) were recorded. Only few patients (*n* = 32, 4.4%) received no drug, whereas 231 patients (31.7%) received at least 5 drugs (i.e., Rx polypharmacy). (**B**) Although disease-modifying drugs for MS accounted for only 21.8% of all prescriptions, they contributed to 92.1% of the total medication costs. (**C**) Cost distribution, with each vertical segment representing one patient, ordered by increasing annual medication cost. Dashed vertical lines indicate the 5th, 25th, 50th, 75th and 95th percentiles. The median medication costs for the patients amounted to €10,994 per year. The medication costs exceeded €35,000 for 7 patients.

Only 32 of the 728 patients (4.4%) had not been prescribed any long-term medication. On the other hand, 231 patients (31.7%) received 5 or more medications and thus had Rx polypharmacy. Two patients were even taking a total of 15 different medications. The average number of Rx drugs taken per patient was 3.7 ± 2.7 (Table 1; Fig. 2A). Most of the patients were treated with a DMD for MS (*n* = 584, 80.2%). The most frequently used DMDs were dimethyl fumarate, fingolimod, glatiramer acetate, interferon β−1a, natalizumab, ocrelizumab, pulsed GCs and teriflunomide (each being taken by > 5% of the patients). Only one patient received a follow-on version of a DMD (Clift, a generic of the non-biological complex drug glatiramer acetate). Overall, the medication plans of the 728 patients contained a total of 2,681 prescriptions, which could be categorized into 21.8% for DMDs, 33.3% for medications to manage MS-related symptoms and 44.9% for comorbidity medications (Fig. 2B).

For most prescriptions (*n* = 2,637, 98.4%), we could find a price information in LAUER-TAXE, but for 110 prescriptions (4.1%), the exact dose was not specified, so that the product with the lowest annual cost was considered. Over the course of a year, the costs for the medication accumulated to a total of €8,581,462, which corresponds to an average of €11,788 per patient. The median annual medication costs were €10,994 (Fig. 2C). DMDs for MS accounted for the largest share of costs (92.1%, mean: €10,858), while symptomatic medications and comorbidity medications accounted for 3.9% (mean: €461) and 4.0% (mean: €468) of the costs, respectively (Fig. 2B). The total medication costs varied greatly among the individuals (SD: €10,601). The highest costs (€66,309) were calculated for a patient who received nilotinib for the treatment of chronic myeloid leukemia in addition to interferon β−1a for RRMS. For some DMDs alone, the costs per year and per patient exceeded €25,000 (IVIg, natalizumab and ocrelizumab, which are all infusion therapies). Injectable interferon β−1a and oral fingolimod followed, with costs above €23,000. The annual costs for the generic glatiramer acetate (Clift, 40 mg three times per week) were comparable to those of the originator product (Copaxone), amounting to €10,381 and €10,923, respectively.

### Factors that are related to individual medication costs

**Fig. 3:**
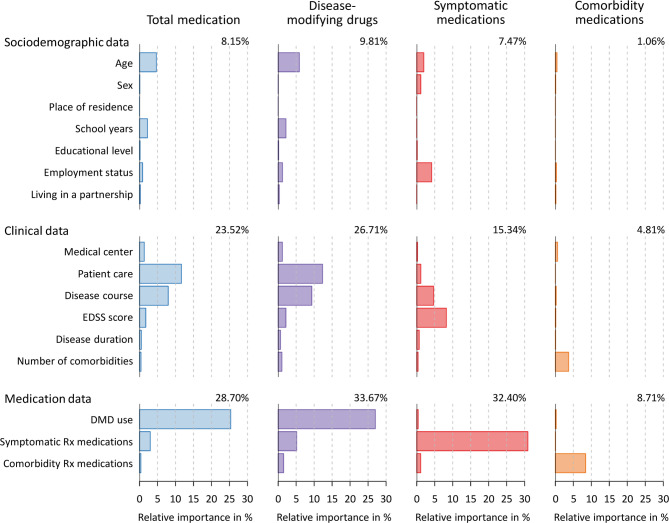
Contribution of patient characteristics to variability in medication costs. The sociodemographic, clinical and medication data of the patients with multiple sclerosis (*n* = 728) were individually fitted to the cumulative annual medication costs using linear models. The bars show the relative importance of each regressor as calculated using the lmg metric [52]. The proportion of variance that is explained by each model is given in the top right corner.

The assessment of the relative importance of sociodemographic, clinical and medication data revealed that the number of Rx medications per category explained 28.70% of the variance in total medication costs (Fig. 3). DMD use was the strongest single predictor with a score of 25.36%, followed by type of patient care (11.63%), disease course (7.96%) and age (4.68%). Outpatients with RRMS who were young incurred higher medication costs than inpatients with progressive MS who were old. The analysis of costs by medication category yielded similar relative importance scores with regard to the costs for DMDs. Regarding the costs for symptomatic medications, the medication data explained 33.67% of the variance, with the number of symptomatic Rx drugs received having the highest relative importance in the model (30.90%). Higher costs for symptomatic medications were also related to higher EDSS scores (8.24%), progressive course of MS (4.70%) and being retired (4.16%). The costs for the long-term medication of comorbidities could not be predicted well. Here, low relative importance scores resulted even for the number of comorbidity medications (8.41%) and the number of comorbidities (3.72%).

**Fig. 4:**
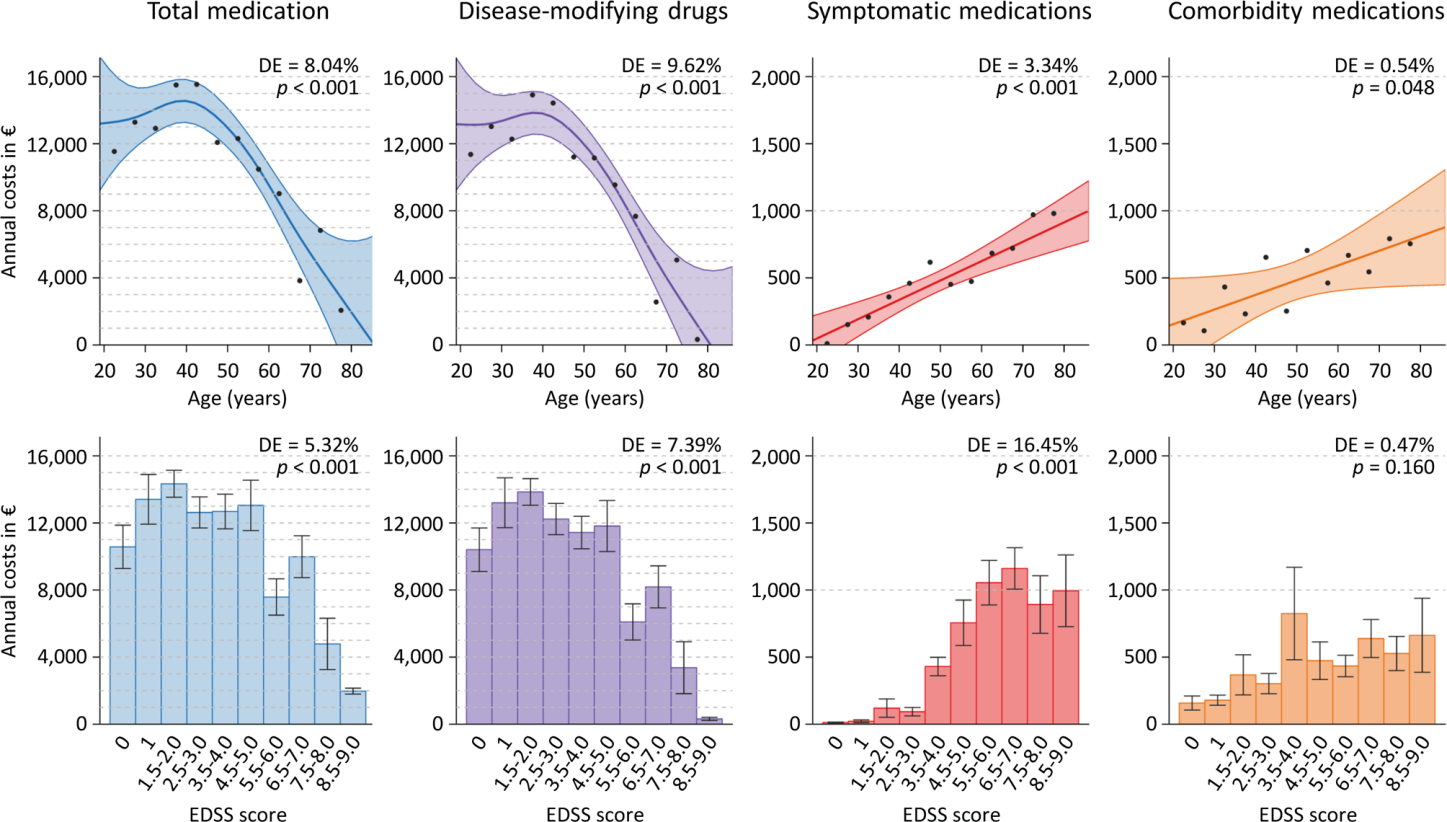
Annual medication costs in relation to the patients’ age and degree of disability. The costs of prescribed long-term medications were calculated for 728 patients with multiple sclerosis. In the upper panels, the average costs for all medications and for each medication category are depicted as points for 5-year age intervals. The smooth curves with 95% confidence intervals were fitted to the data using generalized additive models. The deviance explained (DE) by the models and significance values are specified in the top right corner. The bars in the lower panels show the average medication costs according to the degree of neurological disability as rated using the Expanded Disability Status Scale (EDSS)^37^. Error bars indicate standard errors.

The average annual costs of the patients’ medication are broken down by the categorical variables in Table 2. For men and women, the mean total costs were similar, but the costs of symptomatic medications were significantly higher for men, and the costs for the treatment of conditions other than MS were significantly higher for women. The course of MS was associated with the patient care setting and the employment status, and, in consequence, significant differences in the costs per medication category were found for each of these variables. The distribution of costs by the numerical variables age and degree of disability is shown in Fig. 4. The total medication costs were higher in patients with young age (*τ*=−0.142) or low to moderate EDSS score (*τ*=−0.103), driven by the more frequent use of more expensive DMDs in earlier stages of MS. The costs for DMDs declined sharply after reaching the age of 50 or an EDSS score of > 5.0. Conversely, the annual costs for symptomatic medications and comorbidity medications clearly increased with age and neurological impairment. However, the variability in costs was high and hence the deviance explained by the models was rather small.

**Table 2:** Average annual medication costs (mean ± SD in €) of the patients with MS (*n* = 728) per categorical characteristic.

Characteristic	Total medication	Disease- modifying drugs	Symptomatic medications	Comorbidity medications
Sex			*	*
Women	11,633 ± 10,554	10,729 ± 10,449	395 ± 999	509 ± 2,276
Men	12,172 ± 10,733	11,179 ± 10,860	626 ± 1,132	367 ± 815
Place of residence				
Rural area	11,718 ± 10,086	10,826 ± 10,265	483 ± 1,103	409 ± 1,285
Provincial town	12,240 ± 11,395	11,156 ± 10,526	391 ± 746	693 ± 3,833
Medium-sized town	12,508 ± 9,970	11,600 ± 10,224	438 ± 985	470 ± 1,490
City	11,009 ± 11,291	10,112 ± 11,306	495 ± 1,167	402 ± 1,106
Educational level				
No training	13,427 ± 9,475	13,066 ± 9,554	78 ± 171	283 ± 640
Skilled worker	11,575 ± 10,170	10,670 ± 10,331	463 ± 983	442 ± 1,256
Technical college	11,094 ± 11,463	9,574 ± 10,432	595 ± 1,109	925 ± 4,405
University	12,708 ± 11,526	12,038 ± 11,515	426 ± 1,255	243 ± 575
Employment status		*	***	***
Employed	13,171 ± 10,205	12,582 ± 10,274	232 ± 887	357 ± 1,577
Unemployed	12,790 ± 9,808	11,793 ± 9,948	376 ± 1,136	621 ± 1,332
Retired	10,615 ± 11,031	9,343 ± 10,814	694 ± 1,145	578 ± 2,355
Other	11,260 ± 8,701	11,066 ± 8,686	57 ± 151	137 ± 416
Living in a partnership				
Yes	12,003 ± 10,379	11,136 ± 10,562	447 ± 1,008	419 ± 1,284
No	11,219 ± 11,173	10,124 ± 10,558	497 ± 1,133	597 ± 3,132
Medical center	*			*
Rostock	10,984 ± 10,672	10,102 ± 10,861	456 ± 1,076	426 ± 1,466
Mühlhausen	12,077 ± 9,990	11,243 ± 9,964	473 ± 976	362 ± 705
Greifswald	14,549 ± 11,112	13,228 ± 10,187	460 ± 1,047	861 ± 4,245
Patient care	***	***	***	**
Outpatient	14,813 ± 10,484	14,034 ± 10,280	332 ± 935	448 ± 2,263
Inpatient	5,441 ± 7,634	4,197 ± 7,659	732 ± 1,197	511 ± 1,145
Disease course	***	***	***	***
CIS/RRMS	14,564 ± 10,394	13,951 ± 10,052	219 ± 746	394 ± 2,258
SPMS	5,806 ± 8,135	4,289 ± 8,141	984 ± 1,384	533 ± 1,167
PPMS	7,610 ± 10,013	5,966 ± 10,109	820 ± 1,226	824 ± 1,321
DMD use	***	***		
Yes	14,396 ± 10,213	13,536 ± 10,141	448 ± 1,036	412 ± 1,918
No	1,212 ± 2,390	0 ± 0	516 ± 1,075	696 ± 2,168

* *p* < 0.05, ** *p* < 0.01, *** *p* < 0.001 in Mann-Whitney *U* test or Kruskal-Wallis test.

CIS = clinically isolated syndrome, DMD = disease-modifying drug, MS = multiple sclerosis, PPMS = primary progressive multiple sclerosis, RRMS = relapsing-remitting multiple sclerosis, SD = standard deviation, SPMS = secondary progressive multiple sclerosis.

DMD = disease-modifying drug, EDSS = Expanded Disability Status Scale, Rx = prescription.

## Discussion

Chronic and polysymptomatic diseases often require complex treatment regimens in which several medications are prescribed at the same time. However, with the increasing variety of available treatment options, healthcare costs are continuously on the rise, which creates substantial economic challenges for both providers and patients. The present study provides an overview on the range of costs that are incurred for long-term Rx medications in patients with MS. Our analyses were based on a cohort of 728 patients for whom we calculated the annual medication costs using drug prices in Germany in 2024. This enabled us to explore factors associated with the costs for DMDs and for medications to treat MS-related symptoms and comorbidities. Our study therefore provides useful insights into the financial expenditures for drug treatments in MS.

Our patient cohort had a similar profile in terms of age, proportion of women, disease duration, course of disease and degree of disability as MS patients recorded in European registries^53^. However, the patients were enrolled at clinics with neurology departments that are specialized in the treatment of MS patients. For this reason, they only partly correspond to patients who are treated exclusively on an outpatient basis by neurologists in private practices. For example, the proportion of patients with progressive MS is higher in our dataset than in the NeuroTransData outpatient registry (33.7% vs. 20.0%)^54^. This also implies differences in the use of DMDs: At the time of data collection, a relatively high number of our inpatients (*n* = 113), but none of the outpatients, received GCs as repeated pulse therapy (an off-label practice that is no longer encouraged)^31^, at costs of up to €927 per year. In contrast, the outpatients were treated with a broad spectrum of 18 different DMD preparations, with > 5% of those patients receiving dimethyl fumarate, fingolimod, glatiramer acetate, interferon β−1a, natalizumab, ocrelizumab or teriflunomide. The latter drugs are also the DMDs with the highest prescription prevalence according to outpatient claims data of the statutory health insurance in Germany^55^. Among the high-priced therapies (with annual costs exceeding €23,000) were not only newer DMDs, but also long-established DMDs such as interferon β−1a and natalizumab. The DMD utilization pattern decisively influenced the presented estimates and led to considerable variance in the costs per patient and year. Notably, this pattern is not static, as it is modulated by prescribing norms, reimbursement considerations and physician preferences, which are subject to change over time. It should be also emphasized that a relatively high proportion of our patients (71.8%) had at least one comorbidity and that many patients (31.7%) received 5 or more Rx medications. The proportion of MS patients with at least one or more comorbid condition varies across studies in European countries from 34% to 97.5%^56–59^. The estimated prevalence of polypharmacy in patients with MS ranges from 14% to 76.5%^60,61^. Our study is thus in line with the literature, while the wide range of findings can be attributed to differences in study populations and methodologies (e.g., inclusion or exclusion of as-needed or OTC medications in defining polypharmacy).

Over all our patients, the average annual costs for prescribed long-term medications amounted to €11,788 ± €10,601. However, while DMDs for MS represented only 21.8% of the 2,681 Rx drugs in the medication plans, they made up 92.1% of the cumulative medication costs. Symptomatic medications (*n* = 894) and comorbidity medications (*n* = 1,203), on the other hand, accounted for only 3.9% and 4.0% of the costs, respectively. This corresponds to a study that was published in 2023 by the German NeuroTransData MS Registry^24^. In this study, DMDs also caused the majority of costs relative to other medications (65.2% to 98.3%, depending on the patients’ degree of disability). This study also reported other direct and indirect costs, which were not evaluated in our study: In patients with low disability (EDSS score: 0.0–3.5), the mean costs for DMDs outweighed the other costs (€13,621 vs. €5,694), but in patients with high disability (EDSS score: 7.0–9.5), the mean costs for DMDs were much lower (€5,693) compared to the other costs (€52,883)^24^. Thus, the distribution of cost drivers shifts as disability progresses, with the share of indirect costs increasingly outweighing the share of direct costs in total costs^14^. Similar health economic data on MS in Germany were presented in earlier studies by Flachenecker et al. (2017)^25^, Karampampa et al. (2012)^26^ and Reese et al. (2011)^28^. A study across 16 European countries also showed that the costs for DMDs are the main cost determinants early in the disease, while informal care and productivity losses account for a large proportion of the costs in the late phase of the disease^16^. However, resource consumption was found to be influenced by healthcare systems organization and availability of services in each country. Particularly in patients with severe disability, the costs were highly variable between countries depending on the provision of services, with Sweden and Denmark providing the most support^16^. The aforementioned studies relied on information collected by patient questionnaires and considered the costs of MS from a societal perspective. In contrast, we carefully examined the individual medication plans and specifically captured the costs of all prescribed medications, including those for the treatment of comorbidities. Our data demonstrate a high variation in this direct cost element alone, as the use of DMDs and the number and type of other medications taken varies considerably between the patients.

Our analyses revealed age, EDSS score and disease course as clinical-demographic variables that are strongly associated with the total medication costs. The costs for DMDs, as well as the total costs, were on average 2–3 times higher for patients with relapsing MS compared to patients with progressive MS, which reflects the wide availability of effective treatments for CIS/RRMS^1,8^. The costs by disease course also explain the significant association with the type of patient care, given that the proportion of CIS/RRMS patients among inpatients was relatively low (31.9%). We further found an opposite trend for the costs of medications to treat MS-related symptoms and comorbidities, as they were significantly higher for patients with SPMS or PPMS. Of the clinical-demographic characteristics, a higher EDSS score and a higher number of comorbidities were most clearly related to higher costs for symptomatic medications and comorbidity medications, with relative importance scores of 8.24% and 3.72%, respectively. In most of the existing literature, cost estimates were linked to EDSS levels^14^. Several studies across Europe have shown that the costs for the immunomodulatory treatment of MS are lower in patients with an EDSS score ≥ 7.0 as compared to less disabled patients^16,24–26,28,62–64^. However, when including other direct and indirect costs, the costs were 1.4–2.3-fold higher for moderate disability and 1.8–2.9-fold higher for severe disability as compared to mild disability^14^. Accordingly, the transition from RRMS to SPMS was also associated with a significant increase in total costs^29^. It should be noted that advanced disability primarily leads to higher costs outside the healthcare system. In fact, healthcare was found to account for 68%, 47% and 26% of the total costs in MS patients with mild, moderate and severe disability, respectively^16^. This emphasizes the importance of an early intervention with high-efficacy DMDs to halt or delay disease progression so that the patients’ autonomy and ability to work are maintained and the socioeconomic burden of MS is reduced. Regarding drug costs specifically, previous studies have revealed associations with, e.g., patient age^28,65^, disease duration^28,66^, living area^65^, study center^62^ and the severity of symptoms, such as spasticity^67^ and fatigue^28^. Our data complement these studies with up-to-date annual costs for different medication categories and different patient characteristics. The large variability in the costs for individual patients could, however, only be partly explained by the models.

Several limitations of our study should be pointed out: As previously stated, our patients were recruited from specialized neurology departments and therefore may not reflect the broader MS population seen in general outpatient care. Moreover, the focus of our study was on the medication costs and, therefore, we did not capture other direct and indirect costs of illness, such as costs for hospitalization, neurologist consultations, imaging and laboratory exams, rehabilitation therapies, non-medical investments or MS-related productivity loss. For each patient, the entirety of all long-term prescribed medications was considered, but we did not include acute or on-demand medications, for instance as part of a relapse treatment, or OTC medications that are purchased by the patients themselves without a prescription. The reported costs are based on price lists for Germany and hence cannot be readily generalized to other countries. While our cost calculation approach was designed to provide a standardized and careful estimate, it cannot capture the full complexity of real-world prescribing and dispensing practices. Historical price information was not accessible, and therefore the drug costs could not be valued at the time they were incurred (2017–2022). Instead, we relied on official pharmacy retail prices as of May 2024. While this ensured consistency across all patients and medications, it reflects the cost structure as if the prescriptions had remained unchanged until that time. An overestimation of costs could not be avoided, because negotiated rebates between pharmaceutical manufacturers and statutory health insurers are not disclosed in LAUER-TAXE. On the other hand, as we have generally used the lowest available price for the largest pack size, the annual costs are to be understood as a lower estimate. The use of the least expensive drug preparation in the absence of any dosage information can also be considered a bias. An inherent issue of studies on healthcare costs is the presence of large standard deviations. Therefore, although we have taken great care to accurately calculate the drug costs per patient, the average costs are subject to considerable uncertainty. Nevertheless, our data offer valuable insights into which factors are linked to drug expenditures and to what extent. This information may be useful for planning long-term medication management in patients with MS, enhancing treatment outcomes, optimizing cost efficiency and designing sustainable healthcare policies. Preventing or delaying disability progression at an early stage has long-term benefits for both patients and society and thus remains the main goal in the treatment of MS. However, particular attention should be paid to older patients, which are at an increased risk of polypharmacy^33,61^. Regular checks can help ensure that they receive the medications required to manage their health conditions, while avoiding unnecessary medications. A topic for further research concerns generics and biosimilars in the context of DMDs for MS^68,69^. In our cohort, only one patient received a generic DMD. This may be reflective of both conservative prescribing patterns and restricted market availability in Germany, presumably due to uncertainties arising from protracted patent disputes. Nonetheless, the future relevance of follow-on medicines is likely to increase, although challenges remain regarding their integration into clinical practice to achieve cost-saving benefits in the healthcare system.

## Conclusion

In the present study, we explicitly investigated the cumulative costs per year for all long-term prescribed medications in 728 patients with MS. The costs for DMDs significantly outweighed the costs for symptomatic medications and comorbidity medications. However, with increasing age, increasing degree of disability and the transition from RRMS to SPMS, the costs for the treatment of symptoms and comorbidities were rising. The relatively high medication costs for patients in the early phase of the disease who still have low EDSS scores can be justified by the far higher direct non-medical costs and indirect costs for patients with high levels of disability. Therefore, the early initiation of an active immunomodulatory therapy in patients with MS remains fundamental to slow down disease progression and minimize long-term socioeconomic costs. In the future, cost reductions in MS may be possible, e.g., through the development and use of more effective DMDs, the implementation of improved strategies for therapy escalation and de-escalation, the approval of less expensive follow-on drugs, the avoidance of prescription cascades and the more targeted use of complementary therapeutic interventions.

## Data Availability

The datasets generated during and/or analyzed during the current study are available from the corresponding author on reasonable request.

## Abbreviations

CIS: Clinically isolated syndrome
DE: Deviance explained
DMD: Disease-modifying drug
EDSS: Expanded Disability Status Scale
GCs: Glucocorticosteroids
IVIg: Intravenous immunoglobulins
MS: Multiple sclerosis
OTC: Over-the-counter
PPMS: Primary progressive multiple sclerosis
RRMS: Relapsing-remitting multiple sclerosis
Rx: Prescription
SD: Standard deviation
SPMS: Secondary progressive multiple sclerosis
